# MinActionPath2: path generation between different conformations of large macromolecular assemblies by action minimization

**DOI:** 10.1093/nar/gkae421

**Published:** 2024-05-23

**Authors:** Patrice Koehl, Rafael Navaza, Mustafa Tekpinar, Marc Delarue

**Affiliations:** Department of Computer Science and Genome Centre, University of California, Davis, CA 95616, USA; Plateforme de Cristallographie, C2RT, Institut Pasteur, Université Paris Cité, UMR 3528 du CNRS, 75015 Paris, France; Unité Architecture et Dynamique des Macromolécules Biologiques, Institut Pasteur, Université Paris Cité, UMR 3528 du CNRS, 75015 Paris, France; Unité Architecture et Dynamique des Macromolécules Biologiques, Institut Pasteur, Université Paris Cité, UMR 3528 du CNRS, 75015 Paris, France

## Abstract

Recent progress in solving macromolecular structures and assemblies by cryogenic electron microscopy techniques enables sampling of their conformations in different states that are relevant to their biological function. Knowing the transition path between these conformations would provide new avenues for drug discovery. While the experimental study of transition paths is intrinsically difficult, in-silico methods can be used to generate an initial guess for those paths. The Elastic Network Model (ENM), along with a coarse-grained representation (CG) of the structures are among the most popular models to explore such possible paths. Here we propose an update to our software platform MinActionPath that generates non-linear transition paths based on ENM and CG models, using action minimization to solve the equations of motion. The new website enables the study of large structures such as ribosomes or entire virus envelopes. It provides direct visualization of the trajectories along with quantitative analyses of their behaviors at http://dynstr.pasteur.fr/servers/minactionpath/minactionpath2_submission.

## Graphical Abstract

**Graphical Abstract:**
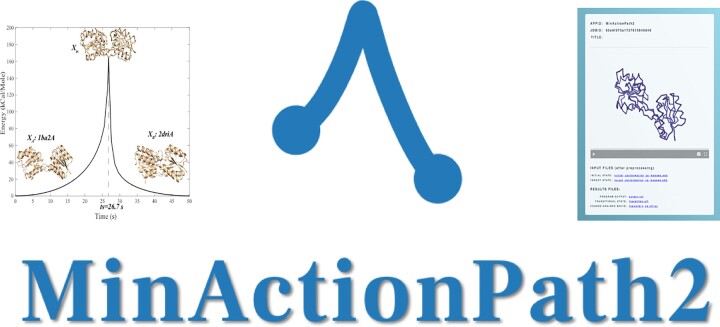

## Introduction

To accomplish their biological function, most of the enzymes and macromolecular assemblies involved in the fundamental processes of a cell cycle undergo a series of conformational changes during their functional cycle (1). For instance, most DNA polymerases switch from an open form to a closed form upon binding the correct incoming nucleotide. This serves as a check point for the enzyme, as this transition occurs only rarely for the incorrect nucleotide and catalysis can occur only in the closed form (2). More complex nanomachines such as the ribosome go through a series of large-scale rearrangements that involve the sliding of the mRNA upon completion of the peptide synthesis step (3). Using cryogenic electron microscopy, it is now possible to generate experimentally the different structures adopted by a given nanomachine in the presence of different substrates. The question remains as to how these conformational changes are achieved and which transition path is followed.

As transitions between allosteric conformations occur in the range of 1 μs–1 ms, capturing experimentally transient structures at the atomic level along their trajectories is a difficult task. The knowledge of such structures, however, would considerably enlarge the scope of structure-inspired drug design. For example, drugs could be designed to prevent the transition in many more ways than permitted by the knowledge of just the endpoints of the transition. In addition, knowledge of the structure of the transition state itself would reveal how exactly catalysis is achieved by a given enzyme.

While computational methods can be used to simulate such transitions, it is important to remember that those transitions are intrinsically rare events. As such, sampling exhaustively the conformational space of the molecule of interest is essential, a sampling that is difficult to obtain with traditional molecular dynamics (MD) simulations. Many new methods are being developed to circumvent this problem (4). In practice, a full atom description of large macromolecular nanomachines is currently difficult to achieve and one must resort to coarse-grained models (5–7). Note that several methods have been developed to go from a coarse-grained representation of biological macromolecules back to their full atomic models (8–10).

In this paper, we derive transition paths between two conformations of a macromolecular system that correspond to a minimum of an action functional, governed by the overdamped Langevin equation. At finite temperature, it is known that such transition paths are found as the trajectories that minimize the Onsager–Machlup action functional (11). Finding such minimum action path (MAP) is equivalent to solving a two-point boundary differential equation. The complexity of this problem is directly related to the complexity of the potential energy considered. Many definitions of this potential have been considered, such as mixing two harmonic potentials centered on each structure (7,12). As our goal is to generate transition paths for large molecular systems (in the hundreds of thousands of atoms), we will consider coarse-grained potentials such as those associated with the elastic network model (ENM) (13).

In a preliminary study (14), we proposed MinActionPath, a method for computing the MAP between two conformations of a proteins on a simplified, two-wells free energy surface derived from the ENMs of the two conformations. The energy in each well is computed as a second order Taylor expansion of the corresponding ENM. Using this energy functional, the equations of motion corresponding to the MAP are solved analytically in each well, and continuity conditions at the crossing between the two wells define the transition point. There were, however, limitations to our implementation of this method. It could not handle structures with loosely connected domains, nor could it always maintain correct stereochemistry over the trajectory using a traditional Tirion elastic network (13). The new implementation, MinActionPath2, also referred to as MAP2, provides a solution to those two problems. First, it allows to define the neighbours in the pairwise sums of the energy function using Delaunay triangulation (15), and second, it provides the option of using a Go-like model to define the potential in proteins (16). It has been shown that the use of such potential reduces the number of covalent bonds with unreasonable stereochemistry during the transition (17). The code has been entirely rewritten to enable the study of very large systems and the web interface now offers a user-friendly visualization of the resulting trajectory in the output.

MinactionPath2 is not the only tool currently available to generate transition paths between two conformations of a protein. The software ProDy is a very useful open-source package that is simple to use (18). It is available for download at http://prody.csb.pitt.edu/comd/. It includes coMD, a tool for generating trajectories using normal mode analysis with a Monte Carlo sampling algorithm that selects the modes that guide the initial structure towards the target structure (19). We note that coMD requires that the input files for the end points of the trajectory must contain the same number of atoms and that those atoms are in the same order. iMODS is a web server that allows for the study of protein dynamics using internal coordinates, i.e. dihedral angles. (20). It includes a morphing tool based on normal modes to generate trajectories, akin to coMD, but with the normal modes parameterized with internal coordinates; we note that it is limited by the size in large macromolecules. Recently, the web server eBDIMS, available at https://ebdims.biophysics.se/, proposes to generate transition paths using Brownian dynamics (21). As MinActionPath2, it is based on elastic networks. It is currently limited to medium-size molecular systems and does not handle inputs with different numbers of atoms, two limitations that are relaxed in MinActionPath2.

In the Materials and methods section, we give details on the formalism and the algorithm itself, followed by a description of the web server and the needed input files and parameters. In the Result section, we present 3 examples: a protein-DNA complex, a ribosome and an entire virus shell. A short Discussion outlines the perspectives.

## Materials and methods

### A minimum action path between two conformations

The MinActionPath algorithm was described in details in (14) and (22). Here, we provide a brief overview for the sake of completeness.

Let us consider a molecule with *N* atoms, described by a position vector $X$. Assuming that we are only interested in effects over long time scales, the dynamic behavior of this molecule can be described by the overdamped Langevin equation.


(1) \begin{equation*}\gamma \frac{{dX}}{{dt}} = - \nabla \left( {U\left( X \right)} \right) + B\end{equation*}

where γ is a friction coefficient, U(X) is the potential energy of the system, $\nabla ( {U( X )} )$ is the gradient of U, and B is an uncorrelated random force with zero mean. By rescaling the unit of time, it is possible to set γ=1. The probability *p* that this equation would have generated a path P over a time interval [0, F] is given by (11)


(2) \begin{equation*}p = \alpha \ exp\left( { - \beta S\left( P \right)} \right)\end{equation*}

where *α* is a normalizing constant,$\ \beta = 1/( {{k}_bT} )$, T is the temperature, *k*_*b*_ the Boltzmann constant, and S(P) the action along the path *P* defined by:


(3) \begin{equation*}S = \frac{1}{2}\mathop \smallint \limits_0^F {\left( {\frac{{dX}}{{dt}} + \nabla \left( {U\left( X \right)} \right)} \right)}^2dt\end{equation*}

Given two possible configurations *X_A_* and *X_B_* for the molecule, the minimum action path that connects these two configurations is the one that maximizes the probability given by Equation (2). This path satisfies the Euler–Lagrange equation:


(4) \begin{equation*}\frac{{{d}^2X}}{{d{t}^2}} = \ \nabla \nabla \left( {U\left( X \right)} \right)\nabla \left( {U\left( X \right)} \right)\end{equation*}

where $\nabla \nabla ( {U( X )} )$ is the Hessian of the potential energy computed at *X*.

In the neighborhood of *X_A_*, the potential is given by a second-order expansion of the potential at *X*_*A*:_


(5) \begin{equation*}{U}_A\left( X \right)\ = \ {\left( {X - {X}_A} \right)}^T{H}_A\left( {{X}_A} \right)\left( {X - {X}_A} \right)\end{equation*}

where ${H}_A( {{X}_A} )$ is the Hessian of the potential energy at position *X_A_*. A similar equation defines the potential in the neighborhood of *X*_*B*_. The total energy *U* is defined as:


(6) \begin{equation*}U = min\left( {{U}_A + \Delta E,\ {U}_B} \right)\end{equation*}

where $\Delta E$ accounts for a difference in free energy between the two configurations *X_A_* and *X_B_*.

The format of the total energy (equation 6) provides a simple strategy for generating a trajectory under Equation (4): (i) find the transition time for which a trajectory starting at *X_A_* under equation (4) based on *U_A_* alone and a trajectory starting at *X_B_* under equation (4) based on *U_B_* alone meet. Note that there are analytical solutions to equation (4) at the left, and at the right of the transition (see 14,22). The point of intersection of the two trajectories needs to be continuous in position, velocity, and energy. We find this point using a conjugate gradient minimizer. It can usually be found in 10–15 iterations. (ii) Once the transition state is known, generate the trajectories on the left (from *X_A_*) and on the right (from *X_B_*) of the transition state.

### Algorithm

The full algorithm to generate the minimum action path between two conformations based on elastic network models has been described elsewhere (14). Here, we describe a modification that has proved essential to enable generating paths for very large molecular systems (22).

From Equations (4) and (5), the equation of motion close to the start conformation *X_A_* is:


(7) \begin{equation*}\frac{{{d}^2X}}{{d{t}^2}} = {H}_A{\left( {{X}_A} \right)}^2\left( {X - {X}_A} \right)\end{equation*}

with the two boundary conditions $X( 0 ) = {X}_A$ and $X( {{t}_s} ) = {X}_{ts}$, where *t_s_* is the transition time and *X_ts_* the transition state. The matrix *H_A_*(*X_A_*) is a real symmetric matrix. As such, it can be diagonalized:


(8) \begin{equation*}{H}_A\left( {{X}_A} \right) = {P}_A{D}_A{P}_A^T\end{equation*}

where *P_A_* is an orthogonal matrix and *D_A_* a diagonal matrix. The solution of Equation (8) is then:


(9) \begin{equation*}{X}_A\left( t \right) = {P}_A{f}_A\left( {{D}_A,t} \right){P}_A^T\left( {{X}_{ts} - {X}_A} \right) + {X}_A\end{equation*}

where ${f}_A( {x,t} )$ is defined as:


(10) \begin{equation*}{f}_A\left( {x,t} \right) = \frac{{sinh\left( {xt} \right)}}{{sinh\left( {x{t}_s} \right)}}\end{equation*}

This approach is limited to macromolecules with less than a few thousand atoms as the diagonalization becomes impractical for larger structures in both computing time and space constraints. There is, however, an alternate approach to diagonalization that is based on the concept of Krylov subspace. For the sake of simpler notations, let us define $A = {H}_A( {{X}_A} )$, $v = {X}_{ts} - {X}_A$, and $f = {f}_A( {x,t} ).$ We build an orthogonal base ${V}_m = [ {{v}_1, \cdots ,{v}_m} ]$ of the Krylov space ${K}_m = \{ {v,Av, \cdots ,{A}^{m - 1}v} \}$ and a tridiagonal matrix T_m_ of dimension *m* such that


(11) \begin{equation*}A{V}_m = {V}_m{T}_m + {\beta }_m{v}_{m + 1}e_m^T\end{equation*}

where *e_i_* is the *i*th unit vector in ${\mathbb{R}}^m$, and ${\beta }_m$is an estimate of the error from this process. It then follows that,


(12) \begin{equation*}f\left( A \right)v \approx \left| {\left| v \right|} \right|\ {V}_mf\left( {{T}_m} \right){e}_1\end{equation*}

Note that *m*, the order of the Krylov space, is taken to be much smaller that the dimension of the matrix *A*, usually in the order of a few tens. In addition, computing ${V}_m$ and ${T}_m$ only requires computing matrix vector multiplications which can be performed efficiently using either the sparse structure of the Hessian *A*, or its tensor representation (23). Finally, the matrix ${T}_m$ is a small tridiagonal matrix of size *m* that can easily be diagonalized.

## Description of the web server

### Initial and final states *X_A_* and *X_B_*

#### Input files

The two input files can be either in PDB format or mmCIF format. Hydrogen atoms and HETATM cards are ignored. There is no limit on the number of atoms or on the number of chains. Chain names are coded with only one character in PDB files, and this is obviously a limitation for macromolecules with more than 62 chains. Chain names using two characters are allowed in the PDBx file format, which is supported in our interface. We found that the best way to handle models with many chains (e.g. in ribosomes or viruses) is to use the mmCIF format with no limitation on the format of the chain name. While mmCIF files are preferred on input, both PDB and PDBx files are accepted and transformed into mmCIF files at this stage. If there are more than one model for each chain, the user can specify to keep only the first one.

#### Choosing the coarse graining

If requested by the user, the program will extract only CA atoms for proteins and C3’ for DNA (or C4’ for RNA); otherwise, the program will use all atoms present in the input files (again, except for hydrogen atoms and ligands identified with a HETATM card). This selection of one specific atom per residue or nucleotide is dictated by the NGL viewer (24). From a simple perspective of mass, however, a nucleotide is on average three times heavier than an amino acid. Users may want to account for this by representing each nucleotide with three atoms. Currently, this needs to be done as a preprocessing prior to using MinActionPath2. Among the three atoms, there should at least be one C3’ atom per residue for DNA (C4’ for RNA) and one CA atom for a protein, to have a correct trace representation of the chains by NGL.

#### Handling a different number of atoms in the two input files

In the previous version of this web site, there was a strict requirement that the two input files contain the same number of atoms. In the new version, we relax this constraint by preprocessing the input files through USAlign (25), with the option –Tmscore 5, which is essentially a sequence alignment, all chains against all chains (option –ter 0). Following this, the common atoms of the aligned residues with the same physico-chemical characters, defined by 5 classes ((P,A,G,S,T), (D,N,E,Q), (H,K,R), (F,Y,W), (V,L,I,C,M)), are extracted and forwarded to the algorithm generating the trajectory itself.

We maintain the possibility for the user to bypass this step, i.e. to generate a trajectory based on the input files without any processing by USAlign. This bypass assumes that the two files have the same number of atoms.

**Figure 1.**
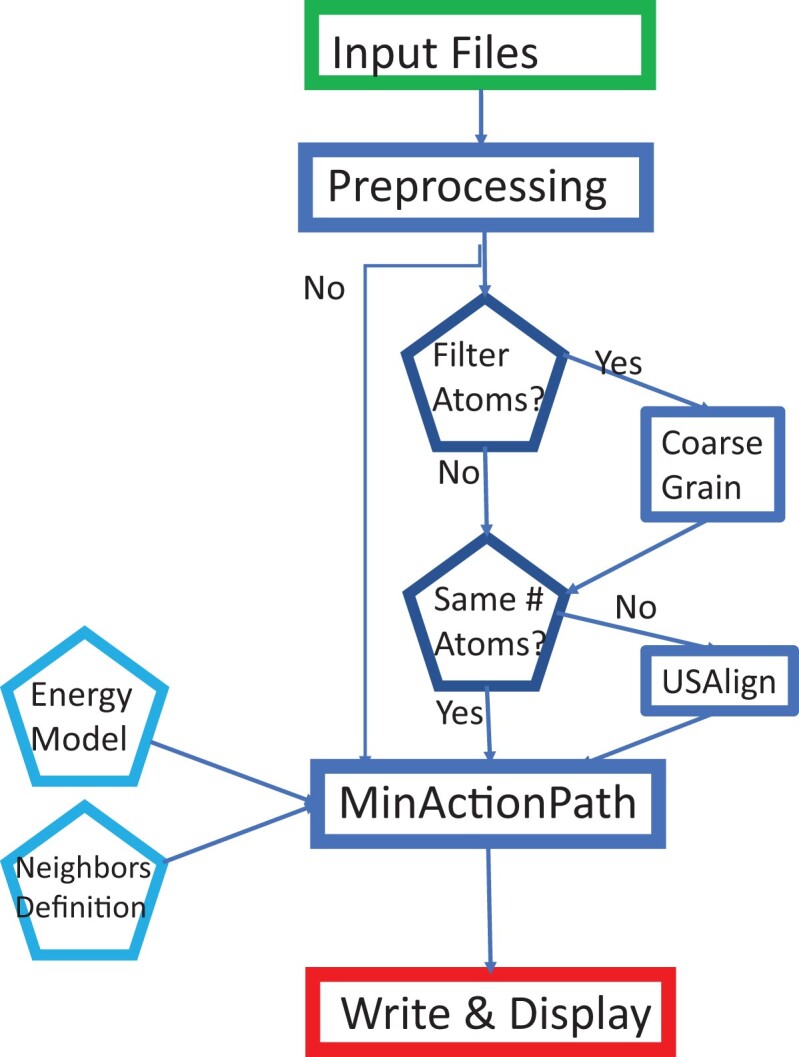
A flowchart of the MAP2 web site.

The general workflow and organization of the web server is summarized in Figure 1.

### The elastic network model

#### Energy

We follow the concept of isotropic elastic network (5,13).

In this model, the energy of a macromolecule is set to be the harmonic energy associated with springs attached to a predefined set of pairs of atoms. Let us consider for example the start conformation ${X}_A$. The energy at a conformation *X* near *X_A_* is set to:


(13) \begin{equation*}V\left( X \right) = \frac{1}{2}{k}_1\mathop \sum \limits_{\left( {i,j} \right)} {\left( {{r}_{ij} - r_{ij}^0} \right)}^2\end{equation*}

In this equation, *k*_1_ is the isotropic force constant of all the springs formed by the pairs of atoms *i* and *j*, ${r}_{ij}$ and $r_{ij}^0$ are the distances between $i$ and $j$ in the conformation *X* and in the reference conformation *X_A_*, respectively, and the summation extends to all pairs (*i*,*j*) included in the network. A similar equation describes the energy of a conformation *X* near *X_B_*, the target conformation, with k_1_ replaced by a different constant *k*_2_. We will refer to this energy as the Tirion energy.

Note that the energy defined by Equation (7) does not account for stereochemistry and as such dynamics that are based on such an energy may undergo too large deviations from acceptable stereochemistry, especially if the two end conformations differ by a large rmsd value. To circumvent this problem, more terms are needed in the energy. The Go Model has been parametrized with this purpose in mind, including terms that are based on bond lengths, torsion angles, dihedral angles, and a van-der-Waals-like term (16,17). Note that the Go model only considers CA atoms and currently works only for proteins. It is available as an option for MinActionPath2.

#### The geometry of the elastic network

Several criteria have been used to define the set of atom pairs that are included in Equation (6). In standard elastic network models, a cutoff distance ${R}_c$ is defined such that all pairs of atoms separated by less than this cutoff are included in the network. There are, however, no guidelines as to which values for ${R}_c$ are best. Typical values for ${R}_c$ are in the range 13–15 Å when the network is based on CA only, and in the range 7–9 Å for all atoms (26). An alternate method is to build a geometric structure on the sets of positions of the atoms; the Delaunay complex is well suited for this purpose (15,27). The advantage of the last criterion is that it is a parameter-free method that handles well possible ‘dangling parts’ of the model, which appear for instance in AlphaFold models for loosely connected domains of large proteins or macromolecular complexes. These two criteria, i.e. a cutoff-based or Delaunay-based elastic network (the default choice), are available in MinActionPath2.

### Choosing *k*_1_ and *k*_2_ for the Tirion energy

In principle *k_1_* can have a different value from *k_2_*: this will influence the position of the transition point, as described in the Results section. The usual way to choose an isotropic *k* value for the Tirion energy is to impose the relationship,


(14) \begin{equation*}k = \frac{A}{{\left\langle B \right\rangle }}\end{equation*}

in kcal/mol/Å^2^ where *<B>* is the average *B*-factor of the structure and *A* is a proportionality factor that contains the temperature-dependence of the elastic constant. The smaller the average *B*-factor, the higher the elastic constant *k*, expected to be in the range 0.16 ± 0.09 kcal/mol/Å^2^.

Note that MinActionPath2 calculates <*B*> for both the start and target structures, if those values are available in the input files, and these values are reported in the log file, so that they may be used to define the ratio of *k*_1_ and *k*_2_ in a subsequent run.

## Output

First the matching of chains found by USAlign is provided, including the sequence alignment of each chain; then the log file of MinActionPath2 is given, which contains details on the convergence of the algorithm as well as a quick analysis of the trajectory in terms of the energy of each frame, its rmsd versus each of the starting and ending states, as well as the *Q*1 and *Q*2 quantities (percentage of specific contacts present in the initial or final state(s), respectively). This allows *Q*1 versus *Q*2 plots to be built; those plots have been found useful when comparing geometrically all sorts of trajectories (14,28). A link to download the mmCIF file of the transition state as well as of the trajectory itself is provided. The entry files to MinActionPath2 (mmCIF) are also provided.

Finally, a window based on the NGL viewer (24) is automatically open on output, where the trajectory can be played, with the possibility to rotate, translate and zoom in the molecule(s).

## Results

MinActionPath2 generates a transition path with minimum action between two conformations of a molecular systems. Its interface has been designed to limit the number of parameters required from the user, to improve the ease of use, without loss of quality. The main inputs include the files containing the start and end conformations for the trajectory, the definition of the elastic networks that represent the structure, as well as the length of time for the trajectory. As described above, the elastic networks can be derived from a cut-off criterium, or by defining a Delaunay complex over the molecule. The energies associated with those networks are based either on a Tirion potential, in which case the user is expected to provide values for the elastic constants associated with the start and target conformations, or a Go-like potential. In the following, we describe a set of worked examples available on the MinActionPath2 web page that are associated with proteins and protein–DNA complexes, as well as brief analyses of the importance of the choices the user needs to make when running our program.

### Worked examples

#### Protein–DNA complex

**Figure 2.**
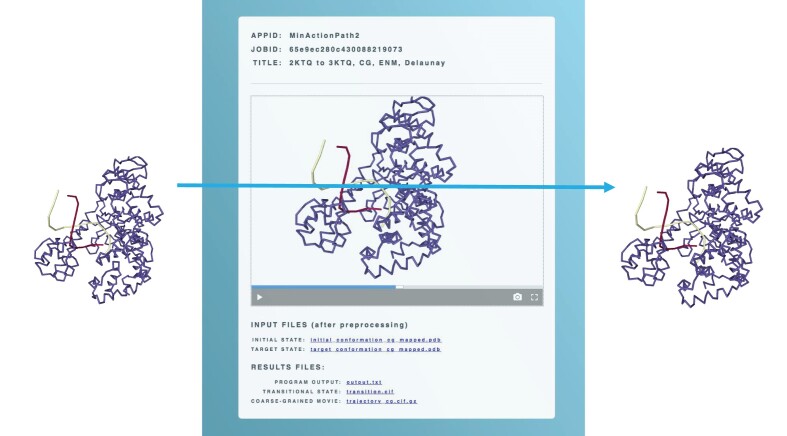
Illustration of the Example 1 with the Klenow fragment of DNA Polymerase I from *E. coli* in complex with DNA.

The first example is the Klenow fragment of DNA polymerase pol I from *Escherichia coli* in a binary or ternary complex with DNA (Figure 2). The input files for the start and target conformations have the PDB codes 2KTQ and 3KTQ, respectively. The two structures have 551 atoms in common when keeping only one atom per residue. The superposition by USAlign allows the extraction of the common residues in the protein (chain A) and in the DNA (Chains B and C, or B and D, which had to be re-matched). The DNA has the same sequence. We use a Delaunay elastic network and the Tirion potential with the default values for elastic constants *k*_1_= 0.1 and *k*_2_= 0.1. Computing the trajectory between conformations 2KTQ and 3KTQ takes only 17 s. This trajectory, and the associated information such as rmsd between snapshots and the initial and final conformations, are available on the MAP2 web server under ‘Examples’. In comparison, computing the trajectory based on all atoms (4401 common atoms) takes 108 s.

#### Ribosome

The second example concerns two conformations of the same ribosome of *E. coli* (8G34.cif and 8G31.cif). Those two conformations contain 52 chains and a total of 10474 atoms in common, after filtering and retaining one atom per residue. The mapping of chains is successfully found by USAlign. We use a Delaunay elastic network and the Tirion potential with the default values for elastic constants *k*_1_= 0.1 and *k*_2_= 0.1. The algorithm finds the transition time in 14 conjugate gradient cycles. The total time to find the trajectory is 263 s. The trajectory is displayed as a movie in the result web page, with each chain colored with a different color. In comparison, computing the trajectory based on all atoms (144 244 common atoms) takes 9125 s. We were able to compute trajectories between eukaryotic ribosomes whose sizes were at least up to 200 000 atoms.

#### Zika virus

We considered two conformations of the entire envelope of the Zika virus, one at pH 5 (PDB code: 5H32) the other at pH 8 (PDB code: 5H37). Both structures represent the virus envelope in the presence of antibody C10 (29). We only retained the coordinates of the CA of the virus envelopes. The corresponding conformations include 180 chains (E proteins) for a total of 71340 atoms after retaining only one atom per residue. We used the Delaunay triangulation to define the geometry of the elastic network. The Tirion potential was set with default values *k*_1_= 0.1 and *k*_2_= 0.1. The algorithm found a trajectory in 1984 seconds. The trajectory and associated information are available on the MAP2 web server under ‘Examples’. Note that in the NGL window, each chain is colored with a different color.

### Influence of *k*_1_ versus *k*_2_ for an ENM model

**Figure 3.**
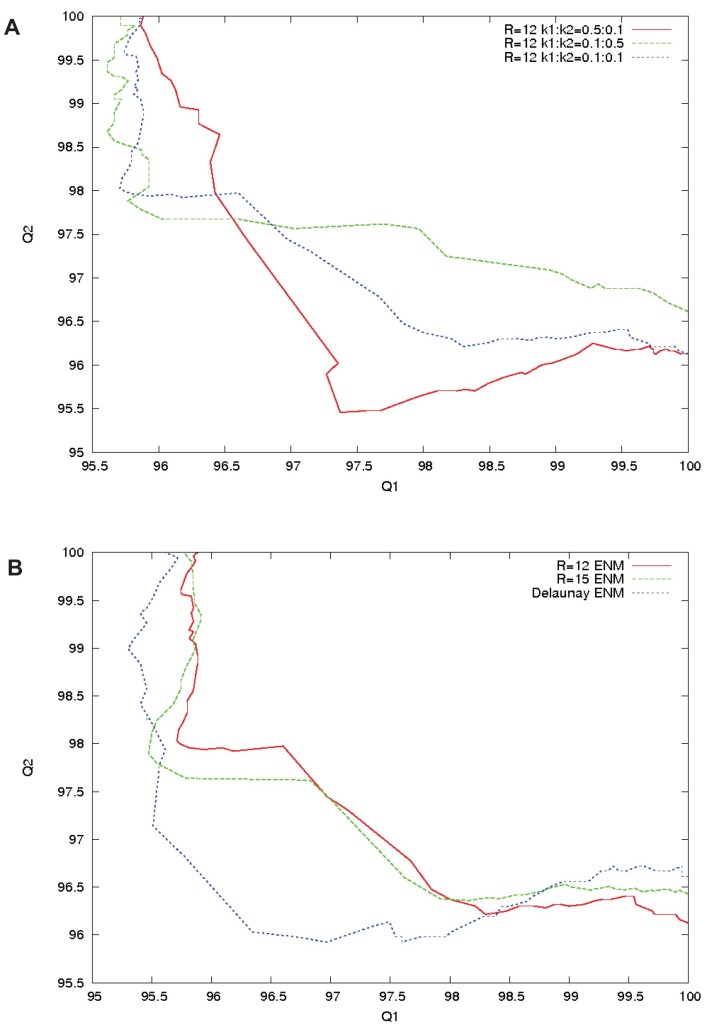
Comparison of the trajectories generated by MinActionPath2 for the pair of structures 1ANF and 1OMP of maltodextrin binding protein. (**A**) *Q*1 versus *Q*2 plots (where *Q*1 and *Q*2 are the percentage of native contacts within the structures of the trajectories with respect to the start and target structures) showing the effect of different elastic constants in the ENM of the initial and final states. (**B**) Same *Q*1 versus *Q*2 plots to compare the effect of using a Delaunay-based elastic network compared with a cutoff-based elastic network.

A test was made to see the influence of choosing different elastic constants *k*_1_ and *k*_2_ for the initial and final states of the molecule of interest. We considered the pair of structures 1ANF and 1OMP of maltodextrin binding protein. Those conformations have 370 residues in common. The rsmd between the start and target conformations is 3.77 Å. As can be seen in Figure 3A, the two *Q*1 versus *Q*2 plots based on *k*_1_= 5*k_2_ and *k*_2_= 5*k_1_ vary above and below the plot *k*_1_= *k*_2_, respectively. For those two settings of *k*_1_ and *k*_2_, the Energies at the transition point are much higher (about 60 kcal/mol) compared to the equivalent energy when *k*_1_ is set equal to *k*_2_ (30 kcal/mol). The transition times when *k*_1_= 5*k_2_ and *k*_2_= 5*k_1_ are shifted to the left, or to the right, respectively, compared to *k*_1_= *k*_2_, as expected.

### Influence of Delaunay- versus cutoff-defined neighbors

In Figure 3B, for the same system of maltodextrin binding protein, we show results obtained with elastic networks defined with a cutoff *R*_c_ set to either 12 or 15 Å, with those obtained with an elastic network obtained from a Delaunay triangulation. The first two cases show similar behaviors, illustrating the fact that similar results are obtained with the cutoff-defined method of defining neighbors provided that *R*_c_> 12, while the latter (i.e. the Delaunay EN) departs more from the diagonal, with a more non-linear behavior. Note that the energies at the transition point cannot be strictly compared as the number of edges in the networks are different.

## Discussion

Action minimization is recognized as an efficient method to generate trajectories described by a Langevin equation (with noise) under the constraint of a known starting and ending points (4), with currently few practical implementations (30). We have described a web site implementation of a faster and more user-friendly version of our initial implementation of the method under a simplified coarse-grained potential (14). The new version, MinActionPath2 can handle large macromolecular assemblies, including entire shells of viruses (>200 000 atoms). It allows for a direct visualization of the trajectory with graphical tools on the output, as well as provides the option of downloading the full trajectory.

The computed trajectory is usually characterized by a very high energy of the transition state, which could appear at first sight as a severe limitation of the program. We note, however, that it is possible to relax this trajectory to improve the energy at the transition, without modifying the overall trajectory significantly (22). We intend to enable such relaxation with MinActionPath2 as future work.

There are many applications that could easily benefit from MinActionPath2. For example, the structures of snapshots along a trajectory between two conformations of a protein can be used to discover new ligand-binding pockets, in very much the same way that normal modes are used to reveal cryptic pockets (31). In addition, the MinActionPath2 trajectories can be used as an input for other methods that requires an initial guess of the trajectory, such as the string methods, for further sampling of possible paths using an all-atom representation and a more physical energy (32,33).

We point out that new methods based on AlphaFold2 are being developed to predict new structures from their sequence, not only in the apo form but also in a holo form (bound to a ligand), with encouraging results (34). Hence, the potential of application of MAP2 is considerably larger than the pairs of similar structures deposited in the PDB.

MinActionPath2 was designed with minimal user inputs in mind, namely the input files for the two structures considered, information on the geometry of the elastic networks (cutoff-based, or Delaunay-based), and information on the energies associated with those networks (Tirion-based with isotropic elastic constants, possibly different for the two input structures, or Go-like potentials that are parameter-free). There is room for improvement with all those choices we have made. First, note that we have chosen isotropic values for the elastic constants associated with the Tirion potential. It is possible to use instead anisotropic elastic constants (i.e. with the option of setting different constants for different pairs of atoms), and even refine those elastic constants based on the *B*-factors associated with the structures (when those B-factors are available). We refer the reader to (23) and (35) for a full discussion on how to choose the elastic constants. We note that in this last case the local elastic constants are set using the surface of interaction between atoms, calculated after Delaunay tessalation: hence, we already have the elements to calculate and calibrate such local elastic constants, both for proteins and nucleic acids (and complexes thereof). Second, our current implementation of the Go-model is parameter free. While this reduces the number of parameters, it may also negatively impact the quality of the trajectory (for example it would not correctly capture difference in the internal dynamics of the two structures that is reflected in the *B*-factor). In future work, we will propose a Go-like potential that can be parameterized. On a side note, our implementation of the Go-like potential is specific to proteins. As a result, the correct stereochemistry may not be maintained during the trajectory, especially in complexes containing nucleic acids. We will parameterize an equivalent potential for nucleic acids and complexes between them and proteins (see for example, (36)).

## Data Availability

MinActionPath2 (MAP2) is free and open to all users at http://dynstr.pasteur.fr/servers/minactionpath/minactionpath2_submission/.

## Notes

Present address: Mustafa Tekpinar, Van Yüzüncü Yil University, Department of Physics, Faculty of Science, Kampus, Van 65080, Turkey.
